# Clinical Use of *Schistosoma mansoni* Antigens as Novel Immunotherapies for Autoimmune Disorders

**DOI:** 10.3389/fimmu.2020.01821

**Published:** 2020-08-13

**Authors:** L. Cleenewerk, Johan Garssen, Astrid Hogenkamp

**Affiliations:** ^1^Division of Pharmacology, Department of Pharmaceutical Sciences, Faculty of Beta Sciences, Utrecht University, Utrecht, Netherlands; ^2^Division of Immunology, Danone Nutricia Research B.V., Utrecht, Netherlands

**Keywords:** *Schistosoma mansoni*, helminths, immune modulation, autoimmune diseases, hygiene hypothesis, M2 macrophages, tolerogenic dendritic cells

## Abstract

The hygiene hypothesis states that improved hygiene and the resulting disappearance of once endemic diseases is at the origin of the enormous increase in immune related disorders such as autoimmune diseases seen in the industrialized world. Helminths, such as *Schistosoma mansoni*, are thought to provide protection against the development of autoimmune diseases by regulating the host's immune response. This modulation primarily involves induction of regulatory immune responses, such as generation of tolerogenic dendritic cells and alternatively activated macrophages. This points toward the potential of employing helminths or their products/metabolites as therapeutics for autoimmune diseases that are characterized by an excessive inflammatory state, such as multiple sclerosis (MS), type I diabetes (T1D) and inflammatory bowel disease (IBD). In this review, we examine the known mechanisms of immune modulation by *S. mansoni*, explore preclinical and clinical studies that investigated the use of an array helminthic products in these diseases, and propose that helminthic therapy opens opportunities in the treatment of chronic inflammatory disorders.

## Introduction: The Hygiene Hypothesis

The incidence of autoimmune diseases, such as inflammatory bowel disease (IBD), multiple sclerosis (MS) and type1 diabetes (T1D) in industrialized countries has continuously increased over the past 50 years, and continues to rise steadily (1, 2). The exact cause of these immune disorders remains unknown, but they are thought to arise as a result of a complex interplay between genetic and environmental factors, leading to immune dysregulation (1). Since genetic changes occur at a slow rate, it is unlikely that the higher incidence of immune disorders over this relatively short period of time is related to a genetic drift. However, there have been substantial changes in environmental conditions (the exposome), including dietary changes, increased pollution, and hygiene that are thought to contribute to the surge in autoimmune disorders (1, 3).

The observation that increasing hygiene in industrialized countries and the resulting low incidence of infectious diseases correlates with an increasing prevalence of allergic and autoimmune diseases, led to the postulation of the hygiene hypothesis in 1989 (4). The hygiene hypothesis states that reduced exposure to pathogens leads to a more reactive immune system, which can result in autoimmunity (1, 2, 4).

**Figure 1:**
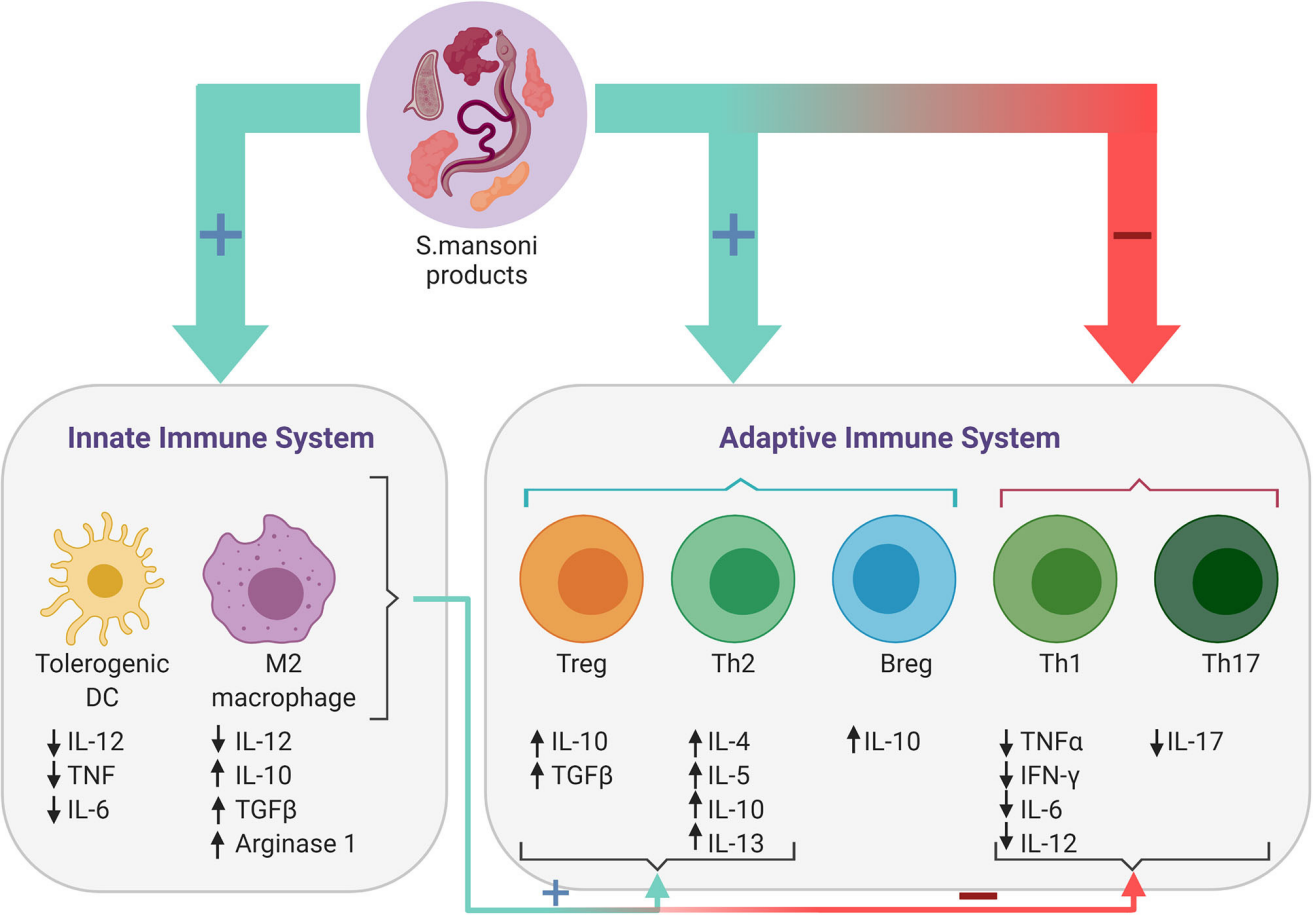
Immune responses induced by *Schistosoma mansoni*. Antigens present on the surface of or secreted by the parasitic worm or its eggs, regulate the host's immune response by modulating both the adaptive and the innate immune response. *S. mansoni* products downregulate Th1 and Th17 responses and reduce levels of the associated pro-inflammatory cytokines, while promoting Th2 and regulatory B- (Bregs) and T-cell (Tregs) responses. Furthermore, *S. mansoni* products also promote the differentiation of tolerogenic dendritic cells (DCs) and alternatively activated (M2) macrophages, which in turn induce Breg and Th2-mediated responses, while simultaneously inhibiting the proinflammatory response of Th1 and Th17 cells.

Bacteria, viruses and parasites have all been implied as players in the hygiene hypothesis (1, 2). These so-called “Old Friends” have co-evolved with humans since the early days of humanity and have been beneficial to our species through their immunoregulatory properties (5). However, with the development of a modern lifestyle, urbanization and increased hygiene, most of these “Old Friends” have been largely removed from our environment. Of particular importance are microbes and parasites that infect humans and induce an asymptomatic carrier state, by inhibiting an inflammatory response (1). In 1970, Greenwood first demonstrated that immunomodulatory properties of parasites can indeed prevent the development of autoimmune diseases in mice by infecting them with *Plasmodium berghei* (6). Parasites such as helminths are particularly well-known for this property (1). Helminths have likely co-evolved with humans, developing the potent ability to induce a state of tolerance in the human body, and fine tuning the immune response to prevent both elimination of the parasite from the body and death of the host from the infection (1). Depending on the species, various mechanisms can induce such a regulatory profile of the immune system, making helminths interesting candidates for new immunomodulatory therapeutics in autoimmune diseases. Despite species-specific differences in the life cycles, tissue tropism and clinical presentation, they are all known to modulate the human host's immune system (7). *Schistosoma (S.) mansoni* is one of the most commonly encountered helminth infections and its immunomodulatory properties (outlined in Figure 1) have been studied extensively (8). We therefore chose *S. mansoni* as a representative member of helminths to explore their mechanisms of immune regulation in autoimmunity. Furthermore, this review provides an overview of the current state of knowledge regarding the use of helminths as a treatment for inflammatory bowel disease, Type I diabetes and multiple sclerosis. To our knowledge, *S. mansoni* has not been used in clinical trials relating to these autoimmune disorders, whereas the use of various other helminthic species (e.g., *Trichuris (T.) suis*) has already been shown to have promising effects. This review therefore also aims to highlight potential mechanistic differences between helminth species, which may provide further insight into the therapeutic potential of *S. mansoni* in helminth-based immunotherapy.

## Schistosoma Mansoni

### History and Epidemiology

Parasites are defined as eukaryotes that use another organism as their habitat (9). Due to the human history of migration, domestication and globalization, which has allowed us to encounter many different parasites, humans are host to over 300 parasite species (9). Parasites have been known to humanity from the beginning of civilization: The Ancient Egyptians were among the first to describe intestinal worms in humans (9). Egypt is also where Theodor Bilharz first identified the helminth *S. haematobium* in 1851 (10). In 1902, Manson discovered another species, *S. mansoni*, the causing agent of intestinal schistosomiasis. *S. mansoni* is responsible for the majority of schistosomiasis cases and accounts for around 300,000 deaths per year (11, 12). Due to under- and misdiagnosis, the number of *S. mansoni* infected individuals likely ranges between 391 and 587 million (13). Because transmission occurs via contaminated water sources, *S. mansoni* is most prominently found throughout the African continent in areas with poor sanitation, with the highest risk of infection in the southern and sub-Saharan Africa and the Nile River valley in Sudan and Egypt. It is also found in several areas in South America, including Brazil, Suriname and Venezuela, and the Caribbean (14).

### Clinical Course of *S. mansoni* Infection

**Figure 2:**
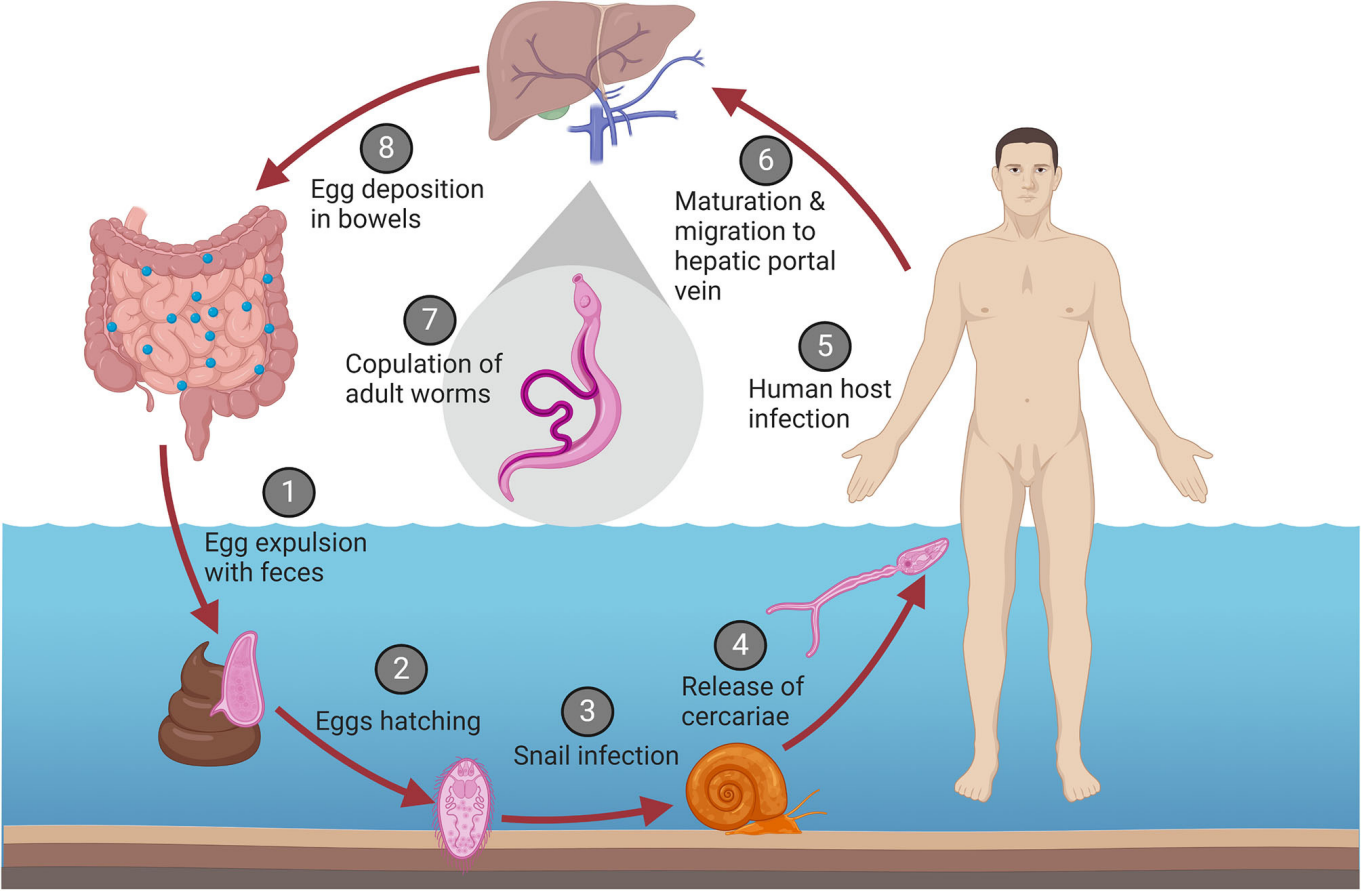
Life cycle of *S. mansoni*. (1) Eggs are excreted with the feces of an infected human host. (2) Under the right conditions in a freshwater environment, the eggs hatch and release the larvae (termed miracidia). (3) The miracidia infect the intermediate snail host, where they develop into sporocysts, asexually replicate, and mature into cercariae. (4) The cercariae are released back into the water. (5) Once the cercariae encounter a human host, they penetrate the skin. (6) After penetration, the cercariae transform into schistosomules and migrate to the hepatic portal vein. (7) The schistosomules mature into adult male and female worms and copulate. (8) The female worm migrates to the mesenteric venules of the bowels and begins egg deposition, that are secreted with the feces and reiterate the cycle.

Infection of a human host is part of the highly complex life cycle of the schistosoma parasites, which is illustrated in Figure 2. This includes sexual reproduction of the adult worms in the human vascular system, an asexual phase in the intermediate snail hosts, followed by a return to a human host after exposure to contaminated water (11, 15). In an infected human host, adult male and female worms copulate in the mesenteric vein. The female worm produces up to 300 eggs daily, approximately half of which are expulsed through the intestinal wall and subsequently excreted with the feces (11, 16, 17). If the excreted eggs reach freshwater and are exposed to suitable environmental conditions, the larvae hatch (18). At this stage, they are termed miracidia, and actively swim using ciliary movements until they encounter the snail intermediate host (11). Once they penetrate the soft tissues of the snail host, the miracidia develop into mature sporocysts (18). Next, the sporocysts reproduce asexually through production of thousands of germinal cells which develop into daughter sporocysts (18, 19), which mature into cercariae that are eventually released from the snail (11). Once the cercariae encounter a human, they penetrate the new host and transform into schistosomules that circulate through the human tissues, lymphatics and venules until they reach the hepatic portal system, where they mature into adult female and male worms, and the reproduction cycle continues (11).

Primary infection usually occurs at a very young age, when children are exposed to contaminated water while bathing or playing. However, acute schistosomiasis is rarely observed in children, most likely due to B and T cell imprinting of children born to infected mothers (11). Therefore, acute infection is most often observed in travelers from non-endemic areas. Since schistosomiasis begins with cercaria entering the skin, the first reaction to infection occurs there, usually within 24–48 h after invasion (20). Dying cercariae in the skin trigger an innate immune response, which leads to a hypersensitivity response and the resulting cercarial dermatitis which presents as urticaria or angioedema (20). Cercarial dermatitis is the result of an inflammatory reaction to a variety of excretory/secretory (ES) proteins that facilitate skin penetration (21).

Late chronic infection causes intestinal disease and hepato-splenic schistosomiasis (15). Chronic infection is established once the mature worms start producing eggs that are then secreted in the stool by the human host. Adult worms do not induce an inflammatory response and therefore do not cause any direct symptoms (15, 22). They are equipped with a variety of strategies that allow them to evade an immune response. In contrast, the eggs are well-capable of inducing an inflammatory response on which they rely to pass from blood vessels into the lumen of the gut so they can be excreted and continue the life cycle (15, 22). However, approximately half of the eggs become trapped in the tissues and attract inflammatory cells, leading to the formation of granulomas and fibrosis. Resulting complications of the chronic infection include organ obstruction, portal hypertension and hepatosplenomegaly with potential gastrointestinal bleeding (11).

### Development of Resistance

Resistance to *S. mansoni* infection is associated with Th2 responses which are characterized by antigen-specific Immunoglobulin (Ig) E, IL-4, and IL-5 production (15). Although it plays an important role in allergic disease, IgE originally developed in response to parasitic infections and provides protection against reinfection with helminths, such as schistosomes (11). While young children mainly produce blocking antibodies, such as IgM, IgG and IgG4, older children and adults predominantly produce the protective IgE, and thus are largely resistant to reinfection (11). The switch from Th1 to Th2 is crucial for survival of the host, indicated by the findings that patients with severe hepatosplenic schistosomiasis have high levels of Th1-associated cytokines [Tumor necrosis factor α (TNFα), IFNγ], while asymptomatic patients exhibit high levels of Th2 associated cytokines (IL-4, IL-5, IL-13) and IgE (23).

The development of resistance against infection with schistosomiasis is slow, and generally takes 10–15 years (15). Children that are regularly exposed to the parasite only show limited resistance between ages 5 and 11. Since the worms do not replicate within the human host, multiple reinfections with schistosomes will eventually lead to a higher worm and egg load. Once the infected individual reaches teenage years, the egg load and the intensity of infection gradually decrease (11).

### Immune Modulation by *S. mansoni*

It is well-known that parasites can induce an immunosuppressive environment to evade the immune system. This also benefits the host, as a reduced inflammatory response limits tissue damage (12). Murine studies have shown that repeated infections with *S. mansoni* lead to the suppression of the immune response, promoting survival of the adult worms (24). The following sections will discuss the early immune response induced by helminthic proteases, and explain the role of dendritic cells, macrophages, and different T-cell subsets herein.

#### Early Immune Responses Induced by Proteases

Proteases are crucial for the survival of parasites. A range of different proteases assist in invasion, nutrient uptake, hatching, evasion of the immune system, and modulation of the host's physiology (25). *S. mansoni* proteases have been shown to regulate vascular functions (25), causing vasodilation, which allows the relatively large adult worms to move more freely to the narrow blood vessels and deposit their eggs there (26). The most well-known *S. mansoni* proteases are cysteine and aspartic proteases, as well as the serine proteases (SP)s (25). The best studied protease, the 28kDa *S. mansoni* cercarial elastase (SmCE) is largely responsible for skin penetration (27, 28). Once cercariae come into contact with the human host, they can enter the skin within 1.5 min, with the help of SmCE (11, 25, 29). SmCE is capable of degrading a large variety of human skin macromolecules (21, 30). Importantly, this protease is also able to elicit an immune response in the host. SmCE induces the production of anti-elastase IgG2a antibodies, which induce macrophage-mediated cytotoxicity against schistosomula and cercariae, resulting in effective killing of the parasite at this stage (31). In addition, both the alternative and classical complement-mediated pathways contribute to the clearance of the parasite during early infection (32, 33).

Although SmCE plays a key role in eliciting this response, it is also involved in resistance against complement-mediated killing (34, 35). During the transformation of cercariae into schistosomula, SmCE assists in remodeling the outer layer of the tegument (i.e., the outer surface of schistosomula and adult worms) and shedding of the glycocalyx which is a potent inducer of the complement system (30, 32, 36).

Next to shedding the glycocalyx, the transforming cercariae remodel the single membrane surface into a complex bi-layer membrane structure, incorporating different host molecules, multi-layered vesicles and glucose transporters (28, 37). Interestingly, the outer surface of the bi-membrane structure can adsorb human blood molecules, therefore masking it from recognition of the immune cells (37). However, several tegument proteins are targeted by the immune system, as can be shown by the production of specific IgE against members of the Tegumental allergen-like (TAL) family (38).

Although the early antigens discussed in this section are crucial for evading initial immune responses during and after invasion, they do not actively modulate the immune response. These proteins are attractive candidates for vaccine development, but are not suitable for immunomodulatory therapy. The processes that lead to immunosuppression by *S. mansoni* will be covered in more detail in the following sections, highlighting the role of dendritic cells and macrophages as these cells largely determine whether a Th1 or Th2 dominant immune response will be initiated (1).

#### Dendritic Cells

Dendritic cells (DCs) are crucial for connecting the adaptive and innate immune responses (1). Depending on the stimuli DCs receive, they can adopt either a tolerogenic or an immunogenic activation state, which in turn affects the differentiation of T-cells (1). The maturation of dendritic cells begins with the uptake of an antigen via pattern recognition receptors (PRRs) such as Toll-like receptors (TLRs) and C type lectin receptors (CLRs). PRRs recognize the so-called pathogen-associated molecular patterns (PAMPs) on infectious agents, which leads to internalization of the pathogen (1, 39). Whereas, immunogenic DCs develop in response to “danger” signals in the form of PAMPs, cytokines or other signals from activated T-cells (39), tolerogenic DCs usually arise in response to apoptotic cells or commensal bacteria, in the absence of “danger” signals. These DCs do not exhibit markers of activation such as MHC and CD86 upregulation. The tolerogenic DC induce Th2 and Treg responses, as seen in helminthic infections (1). Importantly, tolerogenic DCs have been shown to prevent the development of autoimmunity (1). Therefore, helminthic products that promote the development of tolerogenic DCs have therapeutic potential for treating autoimmune disorders.

Indeed, certain helminthic products have been found to direct naïve DCs toward the tolerogenic profile by binding to TLRs or CLRs (such as DC-SIGN) (1). In particular, soluble components secreted by *S. mansoni* eggs, called soluble egg antigen (SEA), and egg-derived dsRNA have shown immunoregulatory properties through induction of tolerogenic antigens. SEA comprises all soluble components of the *S. mansoni* eggs, of which only few have been identified and characterized (40). Studies with murine bone-marrow derived DCs have found that the presence *in vitro* of SEA prevents TLR-dependent conventional activation of DCs. The tolerogenic profile of SEA-exposed DCs was confirmed by minimal upregulation of MHC, absence of CD80/CD86 upregulation and lack of Th1 and Th17-type cytokine production, such as IL-6, TNF and IL-12 (41), and maintain their ability to endocytose, which is lost during conventional maturation of DCs (42). To confirm that these unconventional DCs effectively drive a Th2 response, SEA-treated DCs were transferred to mice. Indeed, when murine SEA-treated DCs were transferred into live animals, they induced the differentiation of naïve T-cells into Th2 cells and the production of IL-4, IL-5, and IL-10 (41, 43). Furthermore, the induction of a tolerogenic DC profile by SEA has been found to be dependent on CD40. Although SEA does not upregulate CD40, absence of CD40 leads to failure to develop Th2 responses by SEA-exposed DCs (44).

On a molecular level, SEA has been found to inhibit pro-inflammatory responses by interacting with the nuclear factor κ B (NFκB) family member B-cell lymphoma 3-encoded protein (Bcl3) (40). Klaver et al. showed that the glycosylation of SEA is essential for the Th2-driving of DCs by suppressing lipopolysaccharide (LPS)-induced, TLR-mediated production of pro-inflammatory cytokines. It is still unclear how exactly DCs drive Th2 differentiation after activation by SEA, but it is known that CD40, OX40L and nuclear factor kappa-light-chain-enhancer of activated B-cells (NF-κB1) expression are required (44–46). However, it is certain that unconventional activation profiles of DC induced by SEA can actively promote Th2 response development (39).

Non-SEA components have also been found to induce tolerogenic DCs. One of the tegumental antigens, Schistosoma mansoni protein 29 (Sm29), has been shown to induce tolerogenic DCs *in vitro* (17, 47). Sm29 is located in the tegument of adult *S. mansoni* and constantly exposed to the immune system, which would explain its immunosuppressive characteristics (47). DCs treated with Sm29 exhibited several characteristics of a tolerogenic profile: higher expression of HLA-DR, CD83, CD80, and CD86 as well as of IL-10 and IL-10R, and increased the frequency of CD4+ T-cells expressing the regulatory molecules CTLA4 and CD25 (47). Taken together these findings suggest Sm29 contributes to the differentiation of naïve T-cells into Treg, unlike the SEA that is a potent Th2 inducer (48).

#### Macrophages

*Schistosoma mansoni* also affects macrophage activity. Macrophages can either be activated via the classical pathway (M1) or the alternative pathway (M2). M1 activation occurs in response to TLR ligands or IFNγ, M2 activation occurs in response to IL-4/IL-13. M2 macrophages, in contrary to M1 macrophages, have low expression of IL-12, but high expression of IL-10, TGFβ and arginase 1 (49). M2 macrophages are present in granulomas and have been found to play a key role in the immunomodulation during schistosomiasis. They have anti-inflammatory functions and play a direct role in modulating fibrosis and survival of the host by downregulating inflammation (23, 50). Interleukin-4-inducing principle from Schistosoma mansoni eggs (IPSE/alpha-1), a major component of SEA, is the main driver of M2 differentiation (49). IPSE/alpha-1 binds to immunoglobulins, with a high affinity for IgE (51). Once it binds to IgE bound to FceRI receptors on the surface of basophils, it triggers the release of IL-4 and IL-13 (51), which directly induces the differentiation of monocytes into alternatively-activated-macrophage-like phenotype and inhibits the secretion of pro-inflammatory cytokines by LPS-stimulated monocytes (51).

Next to SEA, the lipid lysophosphatidylcoline (LPC) can also induce macrophage differentiation into an M2 phenotype (50). LPC is excreted by the worm as a degradation product. It has anti-inflammatory properties such as increasing the immunosuppressive function of Treg cells, promoting eosinophil recruitment and stimulating Th2 polarization through Toll-like receptor 2 (TLR2) dependent mechanisms (50). LPC activates peroxisome proliferator-activated receptor gamma (PPARγ), a transcription factor required for M2 polarization, which in turn increases Major Histocompatibility Complex, Class I-Related (MR1), chitinase 3-like 3 (Ym1), IL-10 and TGFβ, but not Nitric Oxide Synthase 2 (NOS2), gene expression, which is characteristic for M2 macrophages (50). Additionally, LPC induces IL-10 production by macrophages (50). Thus, SEA and LPC, amongst others, drive M2 differentiation of macrophages, which contributes to the immune shift to Th2 response observed in schistosomiasis.

#### B-Cells and IgE

The modulation of dendritic cells and macrophages by *S. mansoni* is important as these cells are directly responsible for the switch of a Th1 to a Th2 mediated immune response. However, *S. mansoni* also employs evasion strategies that directly target the adaptive immune system, in particular B cells and IgE. Because IgE does not play a central role in autoimmune disorders, mechanisms used to evade B cell-mediated immunity will only be briefly discussed.

As the source of the IgE which protects the host, B-cells play an important part in the immune response against *S. mansoni* (52). In *S. mansoni* infected individuals, T helper 2 (Th2) responses and their associated characteristics (IL-4, IL-5 cytokines, eosinophilia, and specific IgE secretion) have been associated with resistance against re-infection (15, 53). *S mansoni* has developed mechanisms to interfere with IgE signaling to evade the immune system. *S. mansoni* antigens are able to cleave the surface-bound low-affinity IgE receptor CD23 (54), which could potentially interfere with T-cell activation. Additionally, *S. mansoni* was found to secrete a homologue of soluble CD23 acting as a decoy receptor by binding IgE and inhibiting activation of the high-affinity IgE receptor FcεRI. This in turn prevents degranulation of basophils and mast cells, inhibiting the release of cytotoxic molecules and inflammatory mediators, which usually contribute to killing of the parasite (54). In addition to conventional B cells, a small subset of B-cells, B regulatory cells (Bregs), are also involved in the response to *S. mansoni* (55). Bregs have recently been identified, as a subset of B-cells capable of producing IL-10 (1), which induces Treg differentiation *in vitro* (55). Furthermore, Bregs can downregulate immune responses through direct interaction with effector T-cells (1). IPSE/alpha-1 can induce production of IL-10 in naïve B cells (55) and stimulate the differentiation of Breg cells (56) Additionally, B cells bind SEA and internalize it, leading to a 3-fold upregulation in the production of IL-10 (55). Bregs have been implicated as important players in autoimmunity (57), and helminth infections have been shown to affect their function in autoimmunity, potentially altering the course of disease (58). Therefore, stimulation of these cells by *S. mansoni* products warrants further investigation.

## Helminth Secretions as a Therapy in Autoimmune Disorders: Should the Use of *S. mansoni* be Promoted?

The immunosuppressive capacity of *S. mansoni* has become of interest in the context of autoimmune disorders (1). Since autoimmune disease are characterized by an overactive immune system and predominant Th1 and/or Th17 responses, it seems plausible that infection with these immunosuppressive helminths could potentially be beneficial in preventing or treating autoimmune inflammatory disorders. However, despite the extensive amount of *in vitro* and *in vivo* studies investigating the effects of *S. mansoni* in autoimmunity, there are no clinical trial reporting use of *S. mansoni* products in autoimmune disorders so far. A recombinant protein of the closely related *S. haematobium* has recently entered clinical trials for the use in IBD (59). Based on the data regarding its effects—discussed in the previous and in the following sections—it could be argued that *S. mansoni* is an eligible candidate for future clinical trials.

In addition, immunomodulation is not a characteristic unique to *S. mansoni* (7); other helminths have already been successfully employed in clinical trials, which—in part—paves the way for future studies investigating the potential benefits of *S. mansoni* in autoimmune disorders. Although species-specific differences are evident, the immunomodulatory mechanisms described in the previous sections largely apply to many helminths such as *T. suis*. *T. suis* has been used repeatedly in clinical trials, especially in IBD and MS (60). This soil-transmitted swine helminth species only transiently infects humans in a self-limiting fashion, while still promoting Th2 immune responses. These characteristics are desirable for human trials.

**Figure 3:**
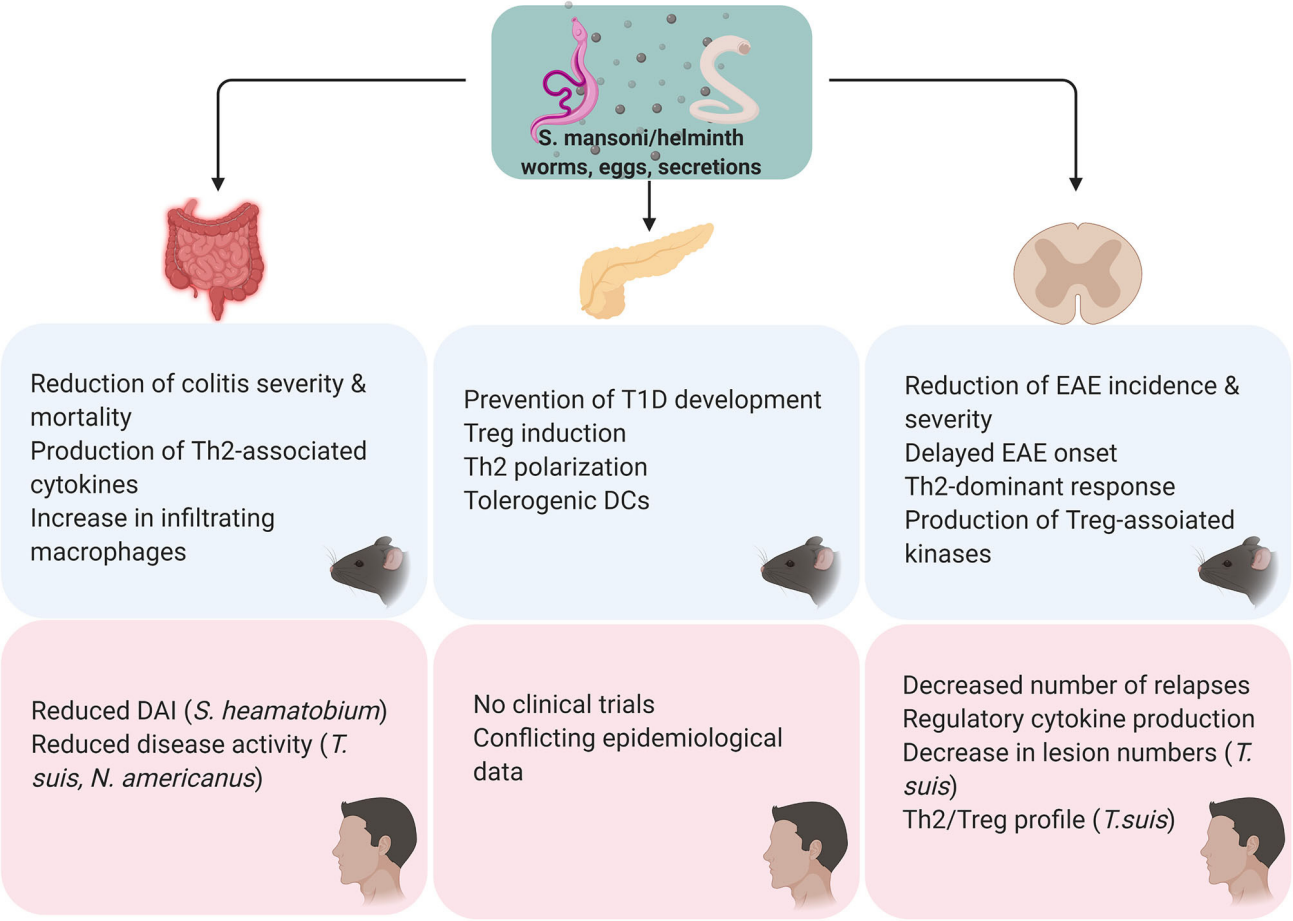
Overview of the findings of S. mansoni/helminth therapy in pre-clinical and in clinical studies. All the *in vivo* studies were performed using *S. mansoni* antigens. For all studies, either cercariae, adult worms, eggs or secretions were used as a therapeutic agent. Preclinical studies in colitis models have found *S. mansoni* therapy to reduce the severity of the disease course, its mortality and modulate the immunological profile by shifting the immune response from a Th1 to a Th2/regulatory profile. Clinical trials using *S. haematobium, T. suis*, and Necator americanus found that helminth therapy effectively improves disease activity. Regarding T1D, preclinical studies in NOD mice showed that helminth therapy inhibits the development of diabetes through the induction of a Th2/regulatory profile and tolerogenic DCs. Clinical studies investigating helminth therapy in T1D have not been conducted and epidemiological data on this subject is conflicting. S. mansoni therapy has been shown to prevent onset and delay the severity of EAE *in vivo*, through a shift from a Th1 to a Th27regulatory profile. Similarly, clinical studies have observed that helminth therapy might be able to reduce relapse frequency and improve lesions via induction of a Th2/regulatory profile.

An extensive study of its excretory/secretory (E/S) proteins has shown that *T. suis* proteins skew DC and macrophage polarization toward a tolerogenic and M2 profile, respectively (60). It has been shown that E/S proteins of *T. suis* inhibit classical activation of DCs, and these DCs skew T cell activation toward a Th2 profile (61). In addition E/S proteins were found to elicit specific Th2 responses, as characterized by the production of IL-4, IL-5, IL-13, and IgE (62). Furthermore, the first transcriptome analysis of *T. suis* uncovered over one hundred potential immunomodulatory proteins (63). Some serine protease inhibitors (serpins) produced by *T. suis* (TsTCI, TsCEI) have been shown to modulate immune responses by inhibiting host proteases, such as chymotrypsin and neutrophil elastase (64). In addition, three novel immunomodulatory proteins (Tsui7583957, Tsui7234544, and Tsui7304731) were identified, but their individual effects have not yet been elucidated (62). However, although several attempts have been made to elucidate the mechanisms of the E/S proteins (65–67), the nature of these components and their exact mechanisms of action are not yet known. The lack of such a large knowledge-gap makes *S. mansoni* an attractive candidate for future studies. Although the nature of the E/S proteins of *T. suis* may be different from *S. mansoni*, the general concepts remain the same (i.e., serine proteases, tolerogenic DC induction). In the following sections, a selection of studies using different helminthic species and/or their products as a therapeutic for IBD, MS and T1D will be discussed, considering them as proof-of-concept studies for the use of *S. mansoni* and its products in the clinic. A summary of these findings is shown in Figure 3.

### Inflammatory Bowel Disease

Inflammatory bowel disease (IBD) encompasses both Crohn's disease (CD) and ulcerative colitis (UC). IBD is characterized by chronic intestinal inflammation leading to irreversible damage of the bowel. While understanding of IBD is still limited, it is believed that CD is mediated largely by Th1 responses, although Th17 responses are emerging more and more as an important contributor to the disease development and progression (68). Furthermore, macrophages and DCs are increasingly recognized as key players in IBD pathogenesis as abnormal activation of these cells leads to inflammatory reactions which contribute to the chronic inflammation. Given their key role in initiating and maintaining inflammation, they are an attractive target for therapeutic agents (69–74). In the developed countries, the prevalence and incidence of CD has risen dramatically since 1940, but this disease is rare in areas where parasitic infections are endemic (75). Taken together, this suggests that helminthic products pose an attractive therapeutic approach.

#### Preclinical Studies

Elliott et al. investigated whether freeze-thaw-killed *S. mansoni* eggs could protect mice from developing trinitrobenzesulfonic acid (TNBS-induced colitis by inhibiting Th1 dependent immune responses (75). Inflammation in TNBS-induced colitis manifests with infiltrating CD4+ T-cells expressing high levels of proinflammatory cytokines, such as IFNγ (75). Histological assessment of the colons showed that mice treated with eggs prior to the TNBS challenge had significantly attenuated colitis and decreased mortality compared to untreated TNBS-exposed mice (75). In mice receiving *S. mansoni* treatment, mesenteric lymph nodes and splenic T-cells produced lower levels of IFNγ and higher levels of IL-4. Furthermore, levels of IL-10 mRNA in the colon were increased in egg-treated mice (75). These findings suggest that treatment with *S. mansoni* eggs inhibits Th1-related inflammatory responses in colitis by inducing regulatory and Th2 responses, which leads to a reduction in symptom severity and mortality (75).

Smith et al. conducted a similar study in which they aimed to determine whether *S. mansoni* infection could protect mice against dextran sodium sulfate (DSS)-induced colitis (76). To determine whether potential protection against colitis was caused by worm or egg antigens, they infected mice either with male worms, resulting in no egg production, or both sexes, causing the release of eggs by female worms. Mice infected with male *S. mansoni* cercariae had a significantly lower disease activity index (DAI), which combines scores for weight loss, bloody feces and stool consistency (76). Additionally, DSS-treated mice presented a shortened colon, while parasite-infected mice had normal-length colons (76). In contrast, schistosome eggs did not provide protection against DSS-induced colitis. Instead, administration of male and female cercariae, which leads to egg production, exacerbated the colitis compared to uninfected mice (76). This observation is likely linked to the inflammation induced by the eggs in the colon to facilitate their passage into the lumen (76). Further results of this study also showed that while IL-10 and TGF-β play protective roles in DSS-induced colitis, the protection provided by male *S. mansoni* cercariae is independent of these cytokines, since treatment with anti-IL-10 and anti-TGFβ did not result in increased symptom scores (76).

Conversely, depletion of macrophages abolished the protective effects of *S. mansoni* and rendered the mice fully susceptible to colitis (76). However, contrary to previous findings, it was demonstrated that the protective effects were not determined by alternatively activated macrophages but instead were mediated by F4/80+ macrophages infiltrating the colon. These macrophages, isolated from schistosome-infected mice, provided protection against DSS-induced colitis if transferred to naive mice prior to DSS exposure (76). Taken together, these results provide evidence that helminthic infections, in particular *S. mansoni* infections, protect against the development of DSS-induced colitis. Additionally, these findings indicate that the protection against colitis is not dependent on the Th1/Th2 axis or Treg cells, but is instead mediated by macrophages, which are not alternatively activated (76).

This latter conclusion is in contrast to the findings by Moreels et al. who suggest that the beneficial effects of *S. mansoni* infection observed in their study were related to an attenuated Th1 response to TNBS, mediated by a shift from Th1 to Th2 profiles In their rat model for TNBS-induced colitis, they demonstrated that disease symptoms and gut inflammation were attenuated in animals with a concomitant *S. mansoni* infection (77). IL-2 secretion in spleens of infected rats was decreased, implying an attenuated Th1 response to TNBS. Moreover, IL-4 levels in the spleen of infected rats were transiently increased, although not significantly (77). Furthermore, the duration of inflammation in response to TNBS stimulation was shorter, and less intense, which correlated with a reduction in inflammatory infiltrates in the colon and faster regeneration of the damaged mucosal layer (77).

The conflicting findings from these studies may be explained by differences in experimental setup. Elliott et al. (75) used dead eggs that were injected, while Smith et al. (76) infected the mice with live worm pairs to induce egg-laying. It is plausible that dead and live eggs would induce different immune responses, and the presence of adult worms in combination with the eggs—in contrast to eggs alone—could also affect the immune response. Mice react differently to *S. mansoni* infections than rats, hence the results obtained by Moreels et al. cannot easily be compared to the outcomes of the murine studies. However, since *S. mansoni* infection attenuated symptoms in all models (75–77), helminth therapy with *S. mansoni* presents itself as a promising approach to preventing onset of IBD. Furthermore, it is likely that both macrophages and Th2 responses are involved in mediating the protective effects of *S. mansoni* although the exact mechanism still needs to be identified.

In a murine model of TNBS-induced colitis, treatment with protein 28 Kd glutathione S Transferase (P28GST) isolated from *S. heamatobium* reduced both clinical and histological scores (78). Levels of pro-inflammatory cytokines (IL-6, TNF, IL-1ß) were significantly downregulated and expression of Th1 and Th17 markers T-bet and ROR-γ, was inhibited. On contrary, an increase in the levels of Arg/iNOS mRNA levels suggested M2 activation of macrophages (78). In a similar study, recombinant schistosome P28GST improved colitis symptoms in rats, which has been related to a Th2 shift of the immune response (79). *S. heamatobium* is closely related to *S. mansoni*, and the P28GST protein expressed by *S. mansoni* has been described to protect against inflammation (80, 81). Thus, proteins with known immunomodulatory properties derived from *S. mansoni* or its close relatives have the potential of being used in the clinic to treat inflammatory disorders.

#### Human Studies

Although the preclinical data suggests that *S. mansoni* protects against the development of IBD, no clinical trials using this particular helminth have been performed so far. However, administration of the recombinant *S. heamatobium* P28GST has been shown safe and effective after administration to 8 CD patients with mild disease in a small recent open label 2a clinical trial (59). Patients received monthly subcutaneous injections of the protein over the course of 3 months and were monitored for 9 months following the treatment. At 3 months after the first injection, disease activity scores decreased by 30% compared to baseline. Furthermore, side effects occurred mostly at the injection site or were possibly related to CD manifestations (59). These promising data suggest P28GST may be used to treat CD, and it would be interesting to assess whether similar results can be achieved with P28GST of *S. mansoni* origin.

Several other helminth species have been used in clinical trials involving IBD-patients. In a clinical trial where *T. suis* eggs were administered to Crohn's patients unresponsive to conventional treatments, it was observed that parasitic treatment might hold therapeutic potential in Crohn's disease (82). A total of 29 patients were enrolled in this 24-week open label study aiming to determine the safety and clinical effects of *T. suis* eggs in Crohn's disease. The patients did not experience any side effects attributable to the infection with the parasite, such as diarrhea, nausea, or abdominal pain. After 24 weeks, close to 80% of patients experienced an improvement in clinical activity (82). Due to the study being open label and the absence of a control group, a placebo effect cannot be excluded. Additionally, the study did not score any other immune parameters such as cytokine levels or immune cell populations, so there is no biological evidence that the results are related to immune modulation. Nevertheless, the results of this study support the hypothesis that administration of helminthic products could benefit patients with IBD, especially if they are non-responsive to conventional treatment (82).

The same group of researchers also conducted a similar clinical trial in patients with active ulcerative colitis (83). In this randomized clinical trial, patients with ongoing, treatment resistant ulcerative colitis ingested *T. suis* eggs every 2 weeks and were assessed for their disease activity scores for 12 weeks following the treatment. A significant improvement in disease activity was seen in 43.3% of the *T. suis* treated patients compared to 16.7% in the placebo group. The treatment was deemed safe, due to the reported side-effects being attributable to other conditions and absence of parasitic reproduction in the human host (83). Although helminthic therapy only induced significant improvement in less than half of the patients, and no data are available as to how the *T. suis* eggs improved symptoms in these patients, results from this study show that it may be beneficial for patients with severe, treatment resistant ulcerative colitis (83). Still, in following studies, it is recommended to measure cytokine levels and other markers of inflammation and disease progression to determine whether the reduction in symptoms is indeed due to the proposed mechanism of action of helminths, i.e., by shifting the immune response to a Th2 profile and thereby reducing the inflammation in the gut.

Intriguingly, ulcerative colitis is believed to be a Th2-mediated disease, which would imply that the Th2 shift induced by parasites exacerbates the disease (84). However, studies have shown that infection with parasites can prevent the development of asthma, which is also Th2-mediated. This effect is believed to be due to helminths regulating the immune system by inducing the secretion of immunoregulatory cytokines, such as IL-10 (85). Even though the exact mechanism is not understood, an improvement in symptoms was observed in patients that were otherwise unresponsive to treatment, without causing any adverse events. Therefore, further investigating the possibility of using helminths as a therapy could lead to the development of a therapeutic agent for some treatment-resistant patients (83).

Apart from *T. suis*, the hookworm *Necator (N.) Americanus* has also been administered to IBD patients in an attempt to reduce the inflammatory response. Unlike *T. suis*, which only transiently infects humans and would therefore require repeated administration, *N. americanus* establishes long-lasting infection (86). In a proof-of-concept study by Croese et al. administration of the hookworm *N. americanus* to five Crohn's patients with long standing but mostly inactive disease resulted in an improvement of disease activity 20 weeks after infection with the helminth (87). However, the attempt to reduce the dose of immunosuppressive drugs following symptom improvement resulted in symptom exacerbation in two patients (87). Similar to the studies mentioned above, these results indicate that helminthic therapy might be a powerful therapy to improve IBD symptoms, at least in combination with conventional immunosuppressive drugs. In the study by Croese et al. the attempt to reduce the dose of immunosuppressive drugs caused the symptoms to exacerbate (87). High doses of immunosuppressants and their side effects might cause problems in combination with parasitic infection. Furthermore, although *N. americanus* does not usually cause pathology, and symptoms are mostly limited to easily treated anemia and itch on the skin penetration site, it can cause an enteropathy (87). Enteropathies were observed in the inoculated patients and although the enteropathy resolved in all five patients in this study (87), it may not be recommended to administer an agent that can cause inflammation in the gut to patients with chronic gut inflammation. The risk/benefit ratio needs to be carefully evaluated in order to provide the best treatment option to every individual patient.

Hookworms and their excretory/secretory products have been studied extensively and their therapeutic properties are reviewed elsewhere (88). *S. mansoni* excretory/secretory products have also been extensively studied, and the immunological effects of these products has been investigated repeatedly. In summary, the promising results of hookworm clinical studies and the knowledge of schistosome immunomodulation provide a steppingstone for further trials using *S. mansoni* and its products as a treatment option in treatment-unresponsive patients.

### Type 1 Diabetes (T1D)

Type 1 diabetes results from an autoimmune response in which CD4+ and CD8+ cells induce destruction of the pancreatic insulin-producing β-cells, leading to insulin deficiency (89, 90). Several studies have provided results that identify T1D as a largely Th1 mediated disease. For example, Katz et al. have shown that Th1 cells that express an autoreactive receptor induced T1D in NOD mice, whereas Th2 cells with the same receptor did not (91). Although the pathophysiology of T1D is not solely dependent on Th1-mediated immune responses, immune modulation toward a more protective immunologic profile could provide protection against development and/or progression of the disease. In respect to this, helminthic products have been shown to modulate the immune response *in vivo* by suppressing Th1-associated immune processes (92, 93). Macrophages and dendritic cells are also key players in the development and pathophysiology of T1D. Their contributions to T1D have extensively been described elsewhere (94–98). In brief, classically activated macrophages and immunogenic DCs contribute to the inflammatory landscape in T1D, whereas M2 macrophages and tolerogenic DCs attenuate the inflammatory response. Thus, modulating the activity of macrophages and DCs by using *S. mansoni* or other helminthic products emerges as a promising therapeutic approach for T1D.

#### Preclinical Studies

Non-obese diabetic (NOD) mice spontaneously develop diabetes (99), which is accompanied by expansion of an autoreactive CD4+ cell population that behaves in a Th1-like manner, infiltration of B-cells, dendritic cells and macrophages into the islets of the pancreas before the development of diabetes-like symptoms (93). Cooke et al. have shown that infection with *S. mansoni* cercariae significantly inhibits the development of diabetes in NOD mice (100). In infected mice, the incidence of diabetes is considerably reduced to 10–15%, compared with 70% of the control mice (100). Furthermore, blood glucose levels in infected mice were demonstrated to be below the cut-off value of 20 mmol/l, which is considered diabetic (100).

Several further studies have shown that helminthic infections and/or products can inhibit the development of diabetes in NOD mice through various mechanisms, such as Th2 polarization, induction of Treg cells and an increase in TGFβ (100–102). Zaccone et al. showed that SEA induces phenotypic changes in murine primary splenic DCs *in vitro*. These phenotypic changes mainly include increased expression of CLRs, such as galectins 1 and 3, and SIGN-R1 (99). Since galectins recognize schistosome antigens and are crucial for the adaptive immune responses induced by the parasite, these results suggest that SEA induces tolerogenic DCs that can inhibit a Th1 response (99). Moreover, SEA was shown to dramatically increase IL-4 and TGFβ mRNA expression in peritoneal exudate cells in mice injected with SEA, compared to controls (99). Furthermore, SEA treatment induced the expression of high levels of IL-4, IL-10 and IFNγ in pancreatic CD4+ T-cells (99). Furthermore, analysis of costimulatory molecules on peritoneal cells and surface markers on peritoneal macrophages suggested the presence of M2 macrophages. Taken together, this demonstrates that SEA-dependent Th2/Treg responses and phenotypic changes in macrophages and DCs may have protective roles in T1D (99). In another study, Zaccone et al. showed that omega-1, one of the main glycoproteins in SEA, drives the differentiation of naïve T cells into Treg cells in DC/T cell cocultures (103).

Altogether, these results show that *S. mansoni* can prevent diabetes in NOD mice (100) and that this protective effect is likely mediated by SEA through modulation of the macrophage and DC activation, leading to regulatory T-cell profiles rather than inflammatory responses (99, 103).

#### Human Studies

Studies aimed at investigating the role of helminths in the human situation are scarce. To our knowledge, the use of *S. mansoni* in the prevention or treatment of T1D has not been studied to date. Epidemiological studies determining the correlation between the incidence of parasitic infections (in general) and T1D are largely lacking, and the ones that have been performed found contradictory results (104). For instance, a study in Southern India found that the prevalence of lymphatic filariasis, a parasitic disease affecting the lymphatic vessels, in patients with T1D was 0%, compared to 2.6% in non-diabetic people, suggesting that parasitic infections protect against T1D (105). However, a large population-based study in Denmark found no correlation between infection with the helminth *Enterobius vermicularis* and the incidence of T1D (106). Evidently, these are entirely different parasites which are likely to induce distinct immune responses. Moreover, parasitic diseases are more prevalent in developing countries such as India (105), which could lead to patients being infected with multiple parasites at once, thus affecting the results of the study. In summary, the role of helminths in T1D remains largely unknown. However, as there are numerous *in vivo* studies that have found strong evidence that helminthic products protect against the development of T1D, it would be worth investigating the potential benefits of treating T1D patients.

### Multiple Sclerosis (MS)

MS is a progressive neurodegenerative disease characterized by gradual loss of mobility, vision and coordination. Worldwide, two million people are affected by this debilitating disease that cannot be cured (107, 108). In MS, chronic inflammation of the central nervous system leads to demyelination and neurodegeneration (109). Similar to T1D, MS is mainly controlled by a Th1-dominated immune response, although other T-cell subsets, such as Th17 cells, and other lymphocytes are also involved (107). Similarly to T1D and IBD, macrophages and DCs play a crucial role in driving the inflammatory process in MS. Skewing of macrophages to a M2 profile and inducing tolerogenic DCs have been proposed as therapeutic interventions (110–114). MS has a higher incidence in industrialized countries than in developing countries, and there is an inverse relationship between helminth infections and MS incidence (108). Thus, treatment with helminthic products presents a promising alternative to conventional treatments.

#### Preclinical Studies

The experimental autoimmune encephalomyelitis (EAE) model is used as the murine equivalent of MS to study the disease. In order to induce EAE, the animals are immunized with a neuroantigen and subsequently develop demyelination and paralysis (108). Similar to MS, EAE is characterized by a strong proinflammatory, Th1-mediated immune response (107). IL-12 appears to be the inducer of the immune disorder in EAE, by activating macrophages and triggering the production of nitric oxide (NO), which is associated with axonal damage and demyelination. High levels of TNFα and TNF-β have also been shown to exacerbate symptoms during relapse in EAE and MS (107). Moreover, an upregulation of Th2-associated cytokines has been associated with recovery from EAE, and adoptive transfer of Th2 cells specific for a neuroantigen has not induced the disease (107).

Infection with live *S. mansoni* cercariae 6 weeks prior to EAE induction significantly decreased the incidence and severity of EAE and delayed its onset (107). Furthermore, the production of the proinflammatory mediators IFNγ, TNFα, and NO was significantly reduced (107). In the spinal cord, levels of IL-12 were significantly reduced in mice infected with *S. mansoni*, suggesting attenuated Th1 induction (107). Additionally, *S. mansoni* infection reduced T cell, F4/80^+^ macrophage, and B220^+^ cell infiltration into the spinal cord (107). Altogether, these results indicate that *S. mansoni* infection protects against the development and progression of EAE, by modulating the immune response, particularly the infiltration of certain inflammatory cell subsets into the CNS (107).

Similarly to cercariae, egg immunization prior to EAE induction resulted in improved clinical scores and delayed onset of the disease (115). Egg immunization 2 days after EAE induction also resulted in delayed onset and decreased clinical scores, while there was no improvement in clinical scores or disease progression if immunization occurs 7–10 days after EAE induction (115). The observed improvements in symptoms and the delay of onset were attributed to reduced CNS infiltration by inflammatory cells and up- and downregulation of IL-4 and IFNγ, respectively (115). IL-10 and IL-5 levels were also elevated in the spleen cells of *S. mansoni* egg immunized mice, which suggested increased Th2 and regulatory responses in these mice (115).

Apart from *S. mansoni*, treatment with helminthic products of S. japonicum (116), *Trichuris suis* and *Trichuris spiralis*, all showed reduction in symptom severity and disease progression related to induction of differential immune activation, including Th2-associated cytokine production and alternative DC activation (14, 116).

In summary, administration of live helminths or helminthic products in EAE animal models appears to reduce the incidence and progression of EAE if administered before the active phase of the disease (61, 107, 115, 116). However, treatment during the active, clinical phase of the disease is not effective. This is likely due to disease progression becoming a self-driving process as a result of extensive inflammation around the lesions, and irreversible tissue damage (108).

#### Human Studies

To our knowledge, no human studies investigating the role of *S. mansoni* in MS have been conducted. However, the potential benefits of helminth infections have been studied. Correale and Farez conducted an observational cohort study during which they followed 12 MS patients with relapsing-remitting MS and concomitant infection with intestinal parasites for over 4 years to investigate whether natural infection with intestinal parasites reduced the number and intensity of symptom exacerbations and changed the immune reactivity (58). MS patients with a parasitic infection were observed to have significantly less relapses; during the study period of 55 months, 3 relapses were observed in the infected group, in contrast to 56 relapses in the uninfected group (58). Moreover, cytokine levels in infected and uninfected patients were measured to determine the effect of the infection on the inflammatory response. Collection and analysis of peripheral blood mononuclear cells revealed an increased amount of IL-10 and TGF-ß secreting cells, and a decreased amount of IL-12 and IFNγ secreting cells in parasite infected patients. There was no difference in IL-4 levels (58). These results indicate that parasitic infections downregulate the inflammatory response in MS.

In a follow-up study involving the same 12 patients, anti-parasitic treatment was demonstrated to change the immunological profile in helminth-infected MS patients and cause symptom exacerbations (117). Anti-parasitic treatment was required in 4 patients that experienced severe symptoms of the parasite infection. and resulted in decreased egg load and reduced levels of IgE, implying successful resolution of the parasitic infections (117). However, the number of exacerbations and the disability score increased significantly in all treated patients (117). Furthermore, anti-parasitic treatment increased the number of new or enlarging lesions in the brain, compared to untreated, parasite-infected controls who presented with a stable number and size of lesions (117). These observations are likely related to the reversal of the previously observed immunomodulation by the parasite. FoxP3+ cells, IL-10, and TGF-β levels decreased significantly, while levels of IL-12 and IFNγ increased after treatment (117). These findings further support the hypothesis that parasites inhibit the progression of MS by inducing a regulatory state of the immune system. Resolution of the infection leads to symptom exacerbation, which is likely due to the removal of the immunomodulatory activities of the parasites (117).

It must be noted that the patients were infected with different parasites, not one specific type of parasite. Even though almost all parasitic infections modulate the immune system and skew the immune response toward a Th2/Treg profile, slight differences in the mechanisms exist (58). Nevertheless, these studies clearly show benefits of parasitic infections on disease progression (58, 117) and support the hypothesis that parasitic infections are in part responsible for the lower incidence of MS in endemic areas (117). Moreover, since the infections with parasites occurred after onset of MS, these studies show that parasitic infections may also be of therapeutic use in patients with ongoing disease, in addition to a prophylactic potential. Unfortunately, if the exacerbation of parasite-related symptoms calls for anti-parasitic treatments, the MS symptoms worsen (117). It is therefore necessary to identify the mechanisms and the antigens involved in the observed immunoregulation, so that treatments that do not require live parasites can be developed.

In contrast to the previous studies, which do not focus on a single parasitic species, Fleming et al. conducted a phase I study in 2011 to determine the safety and potential benefits of administration of *T. suis* eggs to remitting-relapsing MS patients (118). Contrary to a similar study performed in Denmark, which found no clinical efficacy of *T. suis* eggs in MS patients (119), Fleming et al. observed a slight increase in Treg cells, IL-10 and IL-4 in *T. suis* treated patients, and a reduction of the lesions visible in the MRI scans indicating that administration of *T. suis* could have potential beneficial effects for patients with MS (118). Furthermore, oral administration of *T. suis* eggs appeared to be safe, as it resulted in only minor gastrointestinal troubles that spontaneously resolved after a few days (118). This phase I trial was conducted with only a very limited number of patients (*n* = 5), making it difficult to draw any definite conclusions. However, in a recent phase II study following up on the study by Fleming et al. administration of *T. suis* eggs resulted in a decrease of brain lesions, which was accompanied by a decrease in active CD4+ and CD8+ cells, and an increase in the levels of Treg cells and IL-4 expressing cells, implying a shift of the immune response from Th1 to Th2 mediated activity (120). The results of this study are very promising, and will hopefully provide the steppingstone for a phase III trial and the potential to use helminths as therapeutics in the near future.

## Discussion

The increasing incidence of chronic inflammatory disorders such as IBD, T1D, and MS in the industrialized countries is a concerning development, and treatment options are still limited. Helminth therapy has a lot of potential, as it could permanently reprogram the immune system without affecting the response to common infections. Intriguingly, despite its high global prevalence, extensively studied immunomodulatory properties, strong *in vitro* and *in vivo* evidence indicating beneficial effects in a range of autoimmune disorders, *S. mansoni* has not extensively been studied in corresponding clinical trials. To our knowledge, the study reporting on P28GST, derived from the closely related *S. haematobium* describes the only clinical trial involving treatment of an autoimmune disorder with schistosoma (59).

The feasibility of using *S. mansoni* to treat autoimmune disorders is further supported by the observations from clinical trials using other helminth species. These tend to show promising effect, and have generally been found to be safe, although some studies failed to meet primary endpoints or have been terminated for unknown reasons (121). It is important to realize, however, that the immune modulatory properties of helminths vary with species. For example, while *T. trichuria* has been shown to promote the development of inflammatory bowel disease by corrupting the gut epithelial barrier and promoting Th1 responses, *S. mansoni* has a protective effect on inflammation in the gut through promoting the production of IL-10 (2). Obviously, helminth treatment will not be a “one size fits all” therapy. The characteristics of each helminth species need to be carefully studied to provide safe and effective treatments adjusted to the pathophysiology of the disease it would be intended to treat. Only a handful of parasites have been studied in detail, and much of the intricate processes fine tuning the immune response to avoid detection by the host remain unknown. However, although the helminths used in the clinical studies discussed in this review all employ different immunomodulatory strategies, reprogramming of macrophages and DCs is a common feature which supports our hypothesis that *S. mansoni* is a promising candidate for immunotherapy in autoimmune disorders.

Helminth therapies will obviously also have limitations. Despite the promising results, some doubts have been raised concerning the safety and effectiveness of helminth therapy. In this context, the unknown nature of most of the soluble helminthic proteins is a potential drawback. For example, SEA includes a large variety of different proteins, and it is not yet known which (long-term) effects these might have (40). Furthermore, certain helminths have been shown to promote the development of inflammatory disorders (2). Additionally, it has been argued that the decreased incidence of chronic inflammatory disorders such as allergies and autoimmune disease in developing countries is due to under diagnosis, instead of the higher prevalence of helminthic infections (2). However, results from a large number of epidemiological studies investigating the incidence of such disorders in parasite endemic regions, clearly indicate that helminth infections protect against the development of autoimmune, chronic inflammatory disorders. Furthermore, *in vitro* and *in vivo* studies, such as the ones presented in the current review, provide clear evidence that helminthic products modulate the immune system and can induce a state of tolerance, desirable in chronic autoimmune inflammatory disorders.

Another limiting factor in the interpretation of the observed beneficial effects arises from the fact that animal models are not always a reliable representation of the human situation, exemplified by the different responses by mice and rats to *S. mansoni* infections (75, 77). Additionally, even though EAE models have assisted in the development of several effective, now marketed treatments, others that have been effective in animal models have failed in the human situation (108). In particular, a hurdle to the approval of helminth therapy in MS could be the fact that in animal models, EAE could only be prevented, and not treated or cured after onset of the disease (61, 115, 116). This implies that the use of these parasitic products in patients with established disease would not be beneficial. However, a small number of clinical trials in MS patients with established disease did yield positive results, indicating that helminthic infections can protect against disease progression and symptom exacerbation after disease onset (108, 119, 120). Furthermore, the preventive properties could be of interest when people with a genetic predisposition for developing chronic inflammatory disorders are considered.

Despite these limitations, extensive *in vivo* studies using murine models of IBD, T1D, and MS and have found largely beneficial effects of helminthic species on disease incidence and progression, and several clinical trials have given a first glance at the benefits of parasitic treatment for MS and IBD-patients (58, 82, 87, 117, 118, 120). Compared to administering live worms causing chronic infections, protein therapy would be transient and avoid the risk of developing chronic schistosomiasis. Furthermore, in areas with poor sanitation, live helminth administration may lead to transmission of the parasite. Using biologically active and well-defined proteins would abrogate these risks, and circumvents the leniency to undergo helminth treatment as the idea of live worm or egg administration may appear repulsive to patients.

## Conclusion

The idea of using our “Old Friends” as a treatment, or even prophylaxis, opens up a whole new array of potential therapeutics and has the potential to revolutionize the way we treat chronic inflammatory disorders. Based on the studies described in this review, we strongly suggest further study of helminths, especially *S. mansoni*, as an immunomodulatory agent in autoimmune diseases such as T1D, IBD, and MS. Administration of *S. mansoni* proteins in clinical studies could result in the development of new therapeutics without the potential risks of parasite-induced adverse events.

## Author Contributions

LC wrote the manuscript and prepared the figures. AH and JG revised the manuscript. All authors read and approved the final manuscript.

## Conflict of Interest

JG is a Director of Immunology at Nutricia Research, Netherlands. The remaining authors declare that the research was conducted in the absence of any commercial or financial relationships that could be construed as a potential conflict of interest.
